# Effect of Nano-Tricalcium Phosphate and Nanohydroxyapatite on the Staining Susceptibility of Bleached Enamel

**DOI:** 10.1155/2015/935264

**Published:** 2015-07-05

**Authors:** Mohammad Bagher Rezvani, Mohammad Atai, Mohammad Reza Rouhollahi, Kosar Malekhoseini, Hamideh Rezai, Faeze Hamze

**Affiliations:** ^1^Operative Department, Shahed Dental School, Shahed University, Tehran, Iran; ^2^Dental Research Center, Shahed Dental School, Shahed University, Tehran, Iran; ^3^Iran Polymer and Petrochemical Institute (IPPI), Tehran, Iran; ^4^Oral and Dental Diseases Research Center, Kerman University of Medical Sciences, Kerman, Iran

## Abstract

*Objective*. This study was designed to evaluate the effect of nano-tricalcium phosphate (n-TCP) and nanohydroxyapatite (n-HAP) on prevention of restaining of enamel after dental bleaching. *Methods*. Forty bovine incisors were bleached with 20% carbamide peroxide for two weeks. Afterward, they were divided into five groups based on remineralization solution: no treatment (control), 10% n-TCP, 5% n-TCP, 10% n-HAP, and 5% n-HAP. Each group was daily immersed for 10 minutes in the restaining solution (tea) and for 3 minutes in the remineralization agent, respectively. This protocol was repeated for five days. Subsequently, three digital photographs (baseline, after bleaching, and after restaining) were analyzed by Adobe Photoshop software. The obtained *L* ^*∗*^, *a* ^*∗*^, *b* ^*∗*^, and Δ*E* parameters were compared using ANOVA and Wilcoxon and Bonferroni tests. *Results*. After bleaching, there were significant changes in tooth colors (*P* < 0.001) while, after restaining and immersion in remineralization solutions, there were no significant differences in *L* ^*∗*^, *a* ^*∗*^, and *b* ^*∗*^ values of different groups (*P* > 0.05). However, Δ*E* of 10% TCP was significantly lower than the control (*P* = 0.02) while there were no significant differences between the other groups (*P* > 0.05). *Conclusion*. 10% n-TCP could significantly maintain the resultant color and reconstruct the enamel structure after bleaching.

## 1. Introduction

Home bleaching technique has been a popular treatment for discolored teeth [1]. This technique offers an effective treatment which is rather easy, safe, and cost-effective [2, 3]. However, it has some possible side effects [4] such as hypersensitivity, soft tissue burning sensation [5], and recurrent staining of tooth surface in short term [1]. The employed concentrations of carbamide peroxide in home bleaching do not appear to cause any macroscopic changes in surface enamel [6]. However, microscopic alterations such as increased surface roughness and formation of shallow erosions have been reported [2, 6]. These changes in the surface topography are associated with sacrificing the color and glossy appearance of the enamel [7]. The defects mentioned above are the result of a shift in the composition of bleached enamel [8] leading to reduction of calcium, phosphate, and fluoride contents [9]. Since this damage leads to more staining susceptibility after vital bleaching [10, 11], it might be possible to compensate for this problem by employing mineralizing agents [12]. Accordingly, it has been documented that if the enamel surface is recovered with fluoride, casein phosphopeptide-amorphous calcium phosphate (CPP-ACP), or hydroxyapatite (HAP) after bleaching, the microstructural defects might be repaired [7]. Therefore, the bleaching effect would last longer and staining would be prevented [13–15].

Furthermore, it has been discovered that, by reducing the particle size down to nanorange, the remineralization process would be amplified [7]. Indeed, due to the increased surface to volume ratio and proportion of atomicity, the interaction as well as the adhesion of nanoparticles with tooth structure would be improved [10, 16]. On this ground, in recent applications of HAP for biomimetic repair of damaged enamel, it has been confirmed that the nano-HAP (n-HAP) would lead to a considerably superior remineralization [10, 17].

On the other hand, tricalcium phosphate (TCP) is a transitional phase in hydroxyapatite conversion. This complex consists of some structural sites that can be activated by various organic molecules, leading to very good remineralization [18, 19]. TCP has a specific form that overcomes the limited bioavailability of other insoluble calcium phosphates for the remineralization process [20].

Application of bioactive glass or n-HAP in conjunction with carbamide peroxide bleaching has been investigated in very few recent studies [7, 21]. Some of them reported that these complexes do not affect tooth whitening efficacy [21] while others concluded that it would prevent restaining after dental bleaching [10]. It should be noted, however, that the available literature on this object especially comparing the effect of nanoparticles on tooth color stability is extremely scarce. Therefore the aim of this study was to evaluate the effect of nano-TCP (n-TCP) and n-HAP to prevent restaining of enamel surface after dental bleaching.

## 2. Materials and Methods

### 2.1. Materials

Rod-like hydroxyapatite particles (diameter < 100 nm, aspect ratio 2-3) were purchased from Nanoshel Co. (Panchkula, India). Meanwhile, plate-like *β*-TCP nanoparticles (diameter ~ 100 nm) were synthesized in a previous study [22].

### 2.2. Methods

#### 2.2.1. Sample Preparation

Forty caries-free bovine incisors were selected and after cleaning with aqueous slurry of pumice, they were stored in 1% thymol solution. The roots of the teeth were embedded in arch form silicon blocks. Subsequently, a plastic cover was fabricated for the crowns of the teeth using a vacuum forming machine. Finally, a digital photograph was taken in a standard method for determining the color of each tooth.

#### 2.2.2. Photography

All the digital photographs were taken under a standard condition in a complete dark chamber while the distance of the camera (Canon EOS D40) was fixed. In order to work in a constant environment, the background was black while the samples were put in a silicon box. For exposure metering, a circular punch of the gray card with a reflectance value of 18% was put near each sample and the same manual exposure mode was selected for the whole samples [23].

#### 2.2.3. Tooth Bleaching

Carbamide peroxide 20% gel (Opalescence, Ultradent, USA) was inserted in each plastic cover and the crowns of the teeth were exposed to one daily application of the bleaching agents for two hours for fourteen consecutive days. Finally, another digital photograph was taken for recording the color. In order to mimic the oral condition during bleaching, the specimens were stored in 100% relative humidity at 37°C. After each daily treatment, the specimens were thoroughly rinsed with air/water spray and stored in distilled water until the next day.

#### 2.2.4. Experimental Treatment and Restaining Solution

Following the bleaching process, all the specimens were randomly divided into five groups containing eight teeth.

Five 30 × 100 mm glass boxes were prepared and the specimens were immersed in the boxes as follows.

1. The control group specimens (the first group) were daily immersed for 10 minutes in a standard tea solution (boiling 1 gr of tea in 100 mL of water for 2 minutes and then passing the solution through gauze in order to remove the tea leaves), [24] fixed in air for another 10 minutes, and rinsed, respectively. Then they were stored in distilled water until the next day.
2. The second group specimens were daily immersed in 10% TCP for 3 minutes prior to receiving the treatments similar to the control group.
3. The third group specimens were daily immersed in 5% TCP for 3 minutes prior to receiving the treatments similar to the control group.
4. The fourth group specimens were daily immersed in 10% HAP for 3 minutes prior to receiving the treatments similar to the control group.
5. The fifth group specimens were daily immersed in 5% HAP prior to receiving the treatments similar to the control group.

All these treatments were repeated for five days and finally a new photograph was taken for recording the color. Afterward, the photographs were analyzed.

#### 2.2.5. Analysis of Digital Photographs

The Adobe Photoshop software (CS5) was used to analyze the photographs. First, the global color cast of the images was eliminated according to the piece of gray card in the pictures. In order to compare the color, we incorporated the Commission Internationale de l'Eclairage (CIE) system in the form of *L* ^*∗*^, *a* ^*∗*^, and *b* ^*∗*^ obtained by the software. In the CIE system, “*L* ^*∗*^” characterizes the lightness and can range from 0 (dark) to 100 (light). The value of “*a* ^*∗*^” represents the red (+) green (−) spectrum and “*b* ^*∗*^” represents the yellow (+) blue (−) spectrum. Subsequently, *L*, *a*, and *b* values of the selected area were metered and the histogram information was obtained. The Photoshop *L*, *a*, and *b* values were transformed into the CIE *L* ^*∗*^, *a* ^*∗*^, and *b* ^*∗*^ values using the following formulas [23]:

(1) L∗=L×100250,a∗=a−128×240250,b∗=b−128×240250.

Ultimately, Δ*E* ^*∗*^ that represents the total color difference in CIE system was calculated as

(2) $$\begin{array}{c} \Delta E^{*}={\left[\;{\left(\;\Delta L^{*}\;\right)}^{2}{\left(\;\Delta a^{*}\;\right)}^{2}{\left(\;\Delta b^{*}\;\right)}^{2}\;\right]}^{1/2}. \end{array}$$

#### 2.2.6. Surface Morphology Observation

One sample of each group was observed to evaluate the changes in the surface morphology after five days of treatment. The samples were mounted on the aluminum stub using carbon-coated double sided adhesive tape and then coated with gold using a sputter coater. Subsequently, the superficial microstructure of the specimens was analyzed using scanning electron microscopy (SEM) (TESCAN, VEGAII, XMU, Czech Republic).

### 2.3. Statistical Analysis

After exploring the normal distribution, Wilcoxon test was used to compare the baseline color parameters with the results of after bleaching. Meanwhile, the data regarding the specimens receiving mineralizing agents were analyzed using one-way ANOVA and post hoc Bonferroni test. Statistical significance was defined at *P* = 0.05.

## 3. Results

### 3.1. The Effect of Bleaching Protocol

**Figure 1:**
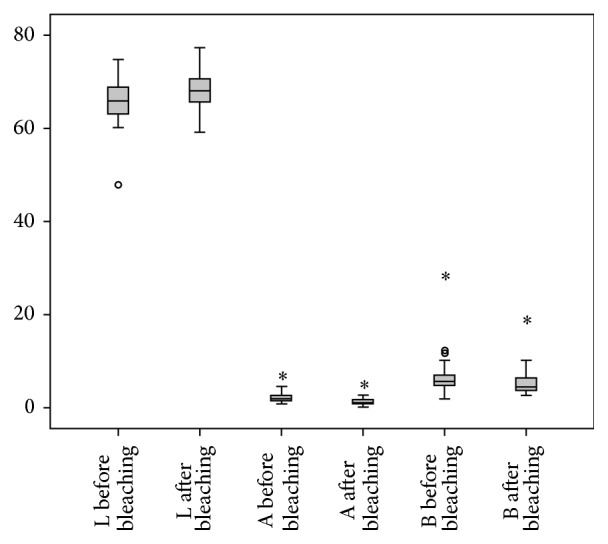
The absolute mean values ± standard deviations of color parameters of the teeth before and after bleaching. *L* ^*∗*^ (lightness) was significantly increased while the absolute amounts of *a* ^*∗*^ and *b* ^*∗*^ (red-green and yellow-blue spectrum, resp.) were significantly decreased. These changes confirm efficient bleaching.

Color parameters of the teeth before and after bleaching (*L* ^*∗*^, *a* ^*∗*^, and *b* ^*∗*^ values) are demonstrated in Figure 1. The baseline *L* ^*∗*^, *a* ^*∗*^, and *b* ^*∗*^ values underwent significant changes after bleaching (*P* < 0.001). Therefore, it was revealed that the bleaching process had significantly improved the enamel color of the examined teeth.

### 3.2. Color Changes Subsequent to Restaining

**Figure 2:**
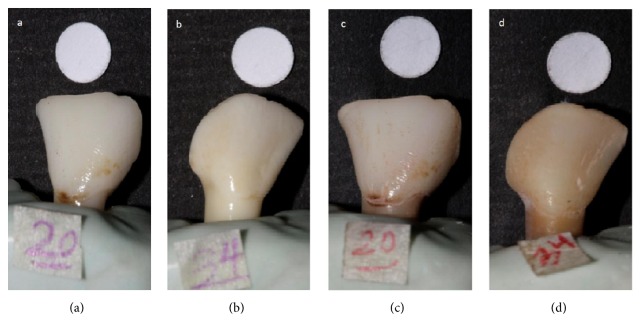
Although the experimental and control samples had quite similar color immediately after bleaching (a and b), the experimental sample that was treated by 10% nano-tricalcium phosphate and restained by tea solution (c) showed more color stability than the control sample (d) that was restained by tea solution without receiving any treatment.

As it is summarized in Table 1, after five days of restaining regimens, there were no significant differences between *L* ^*∗*^, *a* ^*∗*^, and *b* ^*∗*^ values of different groups (*P* > 0.05). However, Δ*E* of 10% TCP was significantly lower than the control group (*P* = 0.02) while there were no significant differences between the other groups, indicating that immersing in 10% TCP solution significantly compensated for the demineralization effect of bleaching process and leading to longer stability of tooth color. Moreover the representative images of experimental and control groups are demonstrated in Figure 2.

### 3.3. Changes in Surface Microstructure

**Figure 3:**
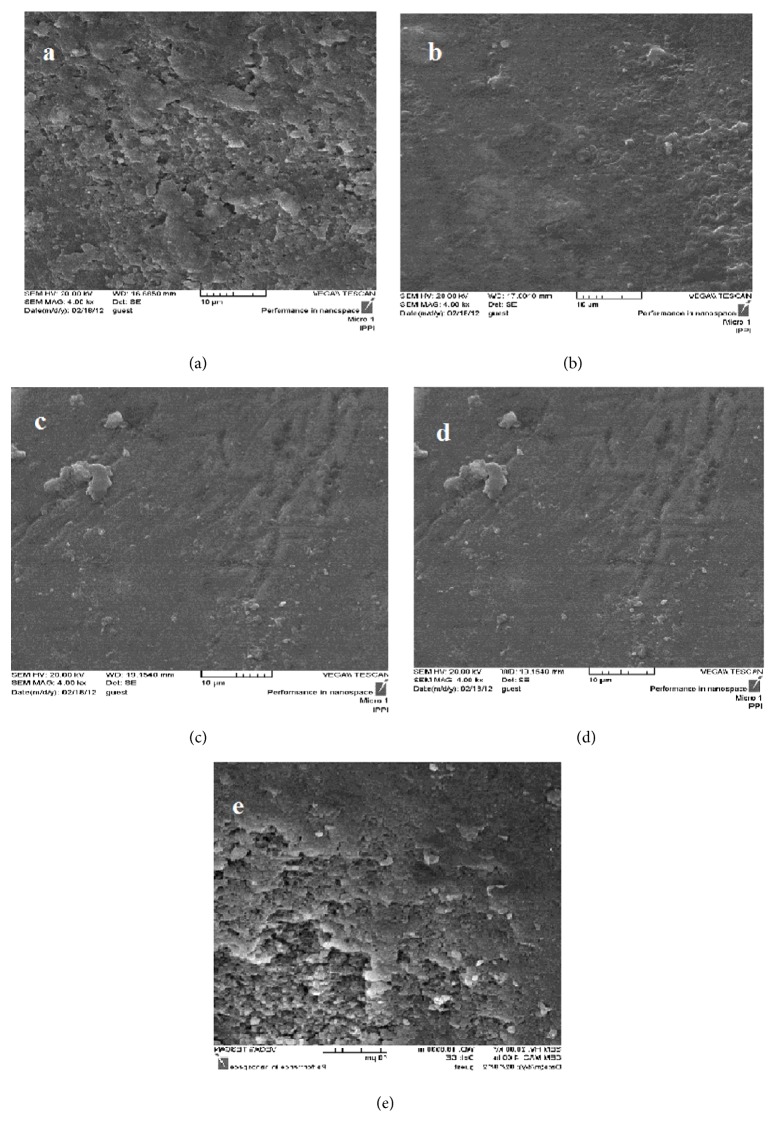
SEM micrographs of the enamel surfaces in different groups (×4000). (a) Control, (b) 10% n-TCP (nano-tricalcium phosphate), (c) 5% n-TCP, (d) 10% n-HAP (nanohydroxyl apatite), and (e) 5% n-HAP. All the experimental group showed smoother surface compared to the control except for the 5% n-HAP.

As Figure 3 illustrates, numerous porosities were observed on the surface of the control and the 5% HAP groups. In contrast, all the samples treated by other remineralizing agents (10% TCP, 5% TCP, and 10% HAP) had considerably smoother surfaces compared to the control. It could be concluded that application of all the studied remineralizing agents, except for the 5% HAP solution, led to an observable remineralization and smoothening of the enamel surface.

## 4. Discussion

The results of the current study showed that application of n-HAP or n-TCP on tooth surface after bleaching protocol would decrease the restaining of enamel. However, only the 10% n-TCP had a significant effect.

The results of the present study are consistent with previous reports incorporating other agents such as fluoride, nanocarbonate apatite, or CCP-ACP after bleaching [10, 15]. This effect is attributed to their mineralizing ability. Accordingly, it has been documented that n-HAP [7] and nanocarbonate apatite [10] penetrate into the intercrystalline spaces and rod sheaths [25, 26]. Therefore, these nanoparticles enhance the superficial enamel smoothness and block up surface defects [7].

Resembling our study, Pedreira de Freitas et al. compared the effect of 2% neutral sodium fluoride and nano-HAP after bleaching treatment and reported that the surface gloss increased only in the nano-HAP group [7]. Since the scattering or reflection of the light strongly depends on the surface texture [7], their investigation could suggest that n-HAP noticeably recovered the surface irregularities caused by bleaching which consequently prevented restaining. Their finding is also in agreement with Singh et al. who reported that the restaining of bleached teeth would be prevented by applying fluoride or CPP-ACP after bleaching [15].

One of the most interesting outcomes of the current research was the stronger effect of n-TCP compared to n-HAP. This finding is in agreement with researchers who studied the soluble compound of calcium and phosphate (amorphous ACP) [27]. They reported that amorphous ACP is more soluble and is more similar to bone or tooth structure compared to crystalline HAP [27]. Therefore, ACP dissolves readily in the oral cavity and redeposits on the damaged enamel [10]. Similar to ACP, the water solubility of TCP is higher than HAP which is quite effective for remineralization [20]. For bone regeneration, the highly crystalline HAP particles are classified as nonresorbable materials while TCP is resorbable [28]. It has been shown that the degradation of biomaterials strongly depends on their solubility [28]. Therefore, the higher solubility of n-TCP could be the reason for its stronger effect compared to n-HAP. This hypothesis has been confirmed by previous publication in which the calcium component of resorbable calcium phosphate materials was introduced as a major factor for local mineralization and also the surrounding calcium pool [28].

However, our results demonstrated a dose-dependent effect for n-TCP because it was not meaningfully effective at 5% but a significant restaining inhibition occurred at 10%. Similarly, in published literatures, dose dependency has been reported frequently for remineralizing agents [29, 30]. It has been documented that, as the calcium content in treatment solution increase, the remineralization would increase too [29, 30].

Moreover, the SEM micrographs showed noticeably smoother surface in the groups receiving remineralizing treatment (except for n-HAP 5%) compared to the control. Accordingly, it has been reported that n-HAP may be deposited onto the enamel surface [31, 32]. Our SEM micrographs demonstrated that many surface defects were produced on the enamel surface as a result of bleaching process, while the nanoparticles reconstructed the surface topography. In a similar study, Gjorgievska and Nicholson applied toothpaste containing bioactive glass on the bleached enamel surface. Their SEM micrographs also represented changes in enamel surface morphology after the bleaching procedure, whereas, by incorporation of the toothpaste, the irregularities were repaired [12].

Although our SEM micrograph showed signs of remineralization in n-HAP groups, our color analysis did not demonstrate significant restaining prevention for n-HAP groups. This finding is consistent with some investigators while it is against some other ones [7, 33]. Pedreira et al. surveyed bleached enamel and claimed significant increase in surface gloss after polishing with n-HAP [7]. This controversy in findings would be attributed to the different methods. The results of this study revealed that restaining by tea solution was strongly prevented by 10% n-TCP. Although in previous researches different solutions were used for restaining, it has been indicated that, compared to coffee or chlorhexidine, teeth have higher susceptibility to staining by tea [24].

Overwhelmingly, as it is shown in Table 1, the results of this study showed that all the treatment solutions decreased the color change compared to the control. However, only 10% n-TCP was significantly effective. Therefore, it can be concluded that some of nanoparticle which has a tooth-like structure would be beneficial for longer efficacy of the bleaching protocol.

## 5. Conclusion

After bleaching, all the experimental solutions prevented the restaining of enamel to some extent. However, only the 10% n-TCP could significantly maintain the resultant color compared to the control, indicating the recovery of the damaged enamel surface by the calcium phosphate compound.

## Figures and Tables

**Table 1:** Bleached and final (after treatment by experimental solutions) values of *L* ^*∗*^, *a* ^*∗*^, and *b* ^*∗*^ for each group and Δ*E*.

Groups	*L*			*a*			*b*			Δ*E*
	Baseline	Bleached	Final	Baseline	Bleached	Final	Baseline	Bleached	Final	
No treatment	67.14 ± 3.14	69.37 ± 2.81	61.21 ± 4.39	−1.69 ± 0.49	−1.00 ± 0.47	−4.12 ± 0.95	−5.48 ± 1.21	−4.20 ± 1.40	−9.92 ± 1.78	12.65 ± 2.38^a^

n-TCP 10%	66.98 ± 3.93	68.76 ± 3.14	64.51 ± 2.92	−2.22 ± 1.10	−1.23 ± 0.71	−3.28 ± 1.44	−5.63 ± 2.59	−4.66 ± 1.93	−6.75 ± 1.95	7.44 ± 1.69^b,c^
n-TCP 5%	66.15 ± 4.08	69.30 ± 3.88	62.37 ± 2.86	−2.19 ± 0.93	−1.48 ± 0.78	−3.91 ± 1.62	−7.36 ± 2.89	−6.26 ± 2.89	−10.24 ± 2.74	11.70 ± 4.07^a,c^

n-HAP 10%	64.59 ± 7.26	66.63 ± 4.80	62.02 ± 3.93	−2.71 ± 3.36	−1.70 ± 1.70	−4.38 ± 1.09	−5.33 ± 0.91	−4.31 ± 0.98	−10.24 ± 3.7	10.25 ± 2.88^a,c^
n-HAP 5%	63.66 ± 1.90	66.56 ± 2.33	60.78 ± 2.97	−2.64 ± 0.82	−1.46 ± 0.46	−3.96 ± 1.02	−7.51 ± 2.72	−6.14 ± 2.00	−9.80 ± 3.16	10.60 ± 4.05^a,c^

Values were mean ± standard deviation. Δ*E* of n-TCP 10% was significantly lower than the control group. Therefore, significant prevention of restaining was achieved in n-TCP 10% group. However, there was no significant difference among other groups.

^a,b,c^Same letters were not significant by Bonferroni multiple comparison of *P* < 0.05. n-TCP indicated nano-tricalcium phosphate. n-HAP indicated nanohydroxyapatite.
